# Pregnancy outcome in women with mechanical prosthetic heart valvesat their first trimester of pregnancy treated with unfractionated heparin (UFH) or enoxaparin: A randomized clinical trial

**DOI:** 10.34172/jcvtr.2020.35

**Published:** 2020-09-05

**Authors:** Minoo Movahedi, Maryam Motamedi, Amirreza Sajjadieh, Parvin Bahrami, Mahmood Saeedi, Milad Saeedi

**Affiliations:** ^1^Department of Obstetrics and Gynecology, Isfahan University of Medical Sciences, Isfahan, Iran; ^2^Department of Internal Medicine, Isfahan University of Medical Sciences, Isfahan, Iran; ^3^Department of Cardiac Surgery, Isfahan University of Medical Sciences, Isfahan, Iran; ^4^Department of General Medicine, Isfahan University of Medical Sciences, Isfahan, Iran

**Keywords:** Low Molecular Weight Heparin, Mechanical Heart Valves, Pregnancy, Unfractionated Heparin

## Abstract

***Introduction:*** Pregnancy increases the risks of thromboembolism for the mother and fetus in patients with mechanical heart valves. The results of some studies have indicated that low molecular weight heparin (LMWH), in comparison with unfractionated heparin (UFH), leads to a lower incidence rate of thrombocytopenia and a decrease in bleeding.

***Methods:*** The present randomized clinical trial involved 31 pregnant women with mechanical heart valves at their first trimester (0-14 weeks) of pregnancy. To perform the study, the patients were divided into two groups, i.e. group A (LMWH group-16 patients) and group B (UFH group-15 patients). The birth weight, mode of delivery, and gestational age at birth as well as the maternal and fetal complications were compared between the two groups.

***Results:*** The mean age of mothers in the UFH and LMWH groups was 32.67±9.11 and 31.50±5.81years, respectively (*P* value > 0.05). Although the rate of maternal and fetal complications was higher in the UFH group as compared with the LMWH group, the observed difference was not significant (*P* value > 0.05).

***Conclusion:*** LMWH can be regarded as a safer therapy for both the mother and fetus due to its lower number of refill prescriptions and fewer changes in the blood level.

## Introduction

Pregnant women with a mechanical heart valve are a high-risk group, for whom an increased rate of maternal or fetal death during pregnancy has been reported. In this regard, some previous studies have reported that only up to 58% of pregnancies were terminated with delivery of live birth.^1^ In fact, pregnancy increases not only the thromboembolic disease risks due to inducing hypercoagulable state but also the fetal risks.^2^

Therefore, it is very challenging and complicated to manage the conditions of pregnant women with mechanical heart valve by selecting the appropriate anticoagulant therapy for the mother that has the least adverse effects for the mother and fetus. Warfarin has been advocated as a proposed anticoagulant in non-pregnant patients with mechanical heart valves^3^; however, as it can cross the placenta and thus impose adverse effects on the fetus by inducing many complications in the fetus, it can even lead to the fetal loss.^4^ Furthermore, there is a risk of bleeding at each stage during the pregnancy. Therefore, the use of heparin as an anticoagulant during pregnancy can be proposed as an alternative strategy to protect the fetus. However, it has been indicated that as unfractionated heparin (UFH) requires frequent injections or continuous infusion and repetitive laboratory control, its prescription leads to pregnant mothers’ lower level of cooperation.^5^ In contrast, low molecular weight heparin (LMWH) has been increasingly used as an anticoagulant in pregnant women; however, limited safety data that addresses its administration has been provided in the literature. Various regimens have been offered to optimize the use of LMWH in pregnant women with mechanical heart valves.^5^

Many studies have also examined the maternal and fetal complications that are caused by the use of these two types of heparin; however, a definitive conclusion regarding the induced complications has not been achieved yet.^6-8^

Given that none of the common therapies proposed for the pregnant mothers is without side effects, and maternal and fetal health is of great significance, further research is required to be performed to offer more appropriate medications with the least complications and the highest therapeutic efficacy. The present study evaluated the incidence rate of maternal and fetal complications following the use of LMWH in comparison with UFH in pregnant women with mechanical heart valves.

## Materials and Methods

The present randomized clinical trial involved all pregnant women with mechanical heart valves at their first trimester (0-14 weeks) of pregnancy that referred to Al-Zahra and Beheshti hospitals in Isfahan, Iran during 2017-2018. At a 95% confidence interval and 80% test power and according to the results of previous studies regarding the incidence rate of 4% thromboembolic events in pregnant women with mechanical heart valves^9^ as well as the error level of 20%, the sample size for each group was considered to be 17 patients that were randomly selected from the available target population.

The inclusion criteria were having a mechanical heart valve in aortic or mitral position, being at the first trimester of pregnancy, lack of hyper sensitivity to any of the studied drugs, and absence of recurrent abortions or frequent stillbirths associated with antiphospholipid syndrome. Moreover, being under the supervision of the researcher from the beginning (rather than the midst) of pregnancy and having class I or II NIYHA were the other inclusion criteria.

**Figure 1:**
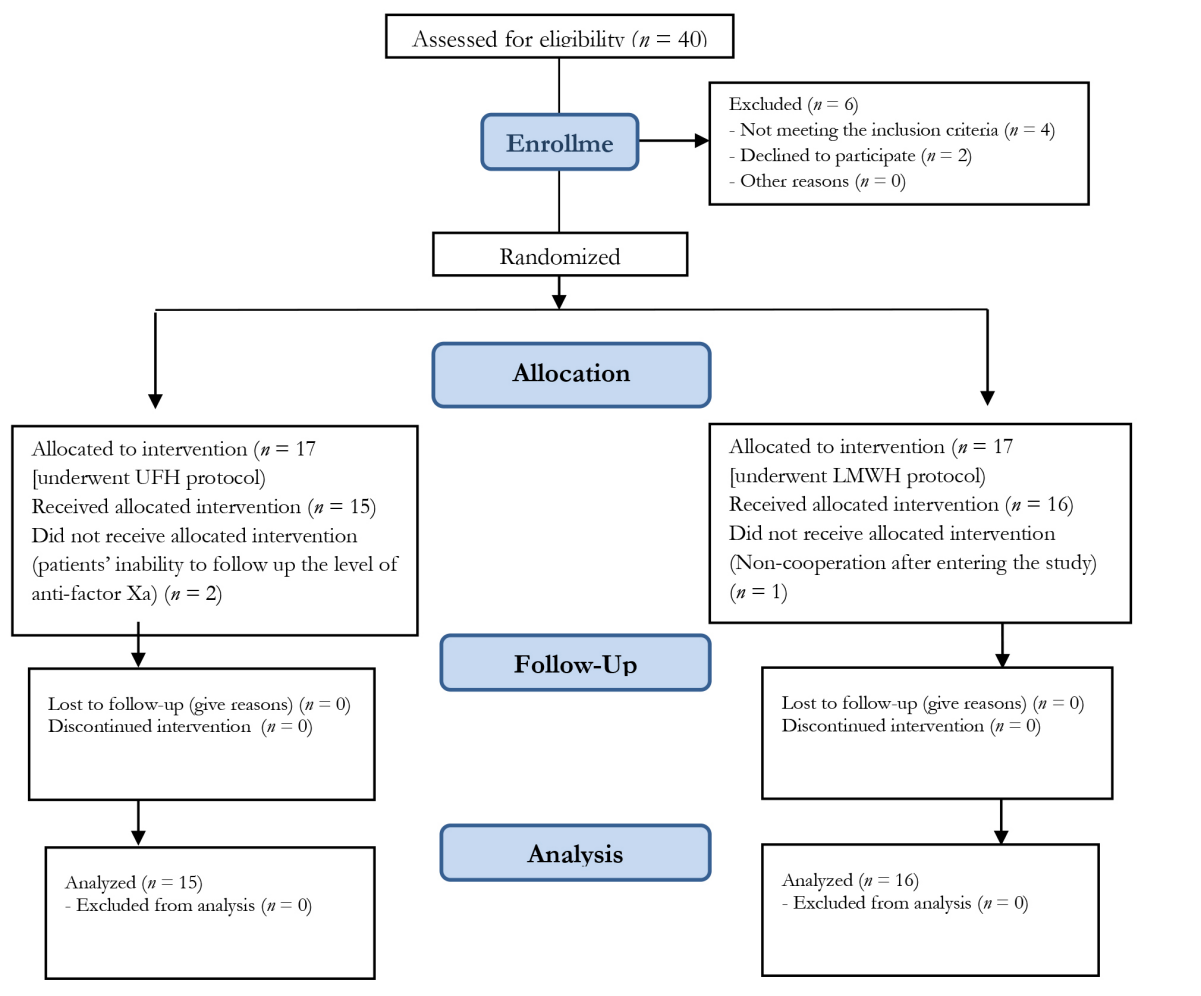
Consort patient flow diagram

Patients’ non-cooperation after entering the study, lack of access to the patient, inability to follow-up the patient’s condition, immediate compulsory termination of pregnancy, the use of warfarin in the first three months of pregnancy, inability of patient to follow up the level of anti-factor Xa, and thrombocytopenia (less than 75,000) were considered as exclusion criteria (Figure 1).

After obtaining the code of ethics from the Ethics Committee of Isfahan University of Medical Sciences (IR.MUI.MED.REC.1397.362) and the code from the Iranian Randomized Clinical Trial (IRCT) (IRCT20171030037093N28) as well as gaining the written consent from the pregnant women eligible for the study, the patients were divided into two groups by the sequencing site (http://sealedenvelope.com) using the randomized block design (with four blocks). At baseline, the patients’ basic information such as age, number of pregnancies, weight, gestational age, and the position of mechanical heart valve, i.e. mitral or aortic were recorded. All patients received bi-leaflet prosthetic valve at the time of surgery.

Subsequently, LMWH was administered to the case group from the beginning of the pregnancy to the end of the first trimester. The common treatment with UFH was used for the control group as follows: UFH, therapeutic dose of warfarin, and UFH were administered in the first, second, and third trimesters, respectively.

The therapeutic dose of all drugs was determined following a consultation with a gynecologist. An initial dose of 80 mg/kg and then continuous infusion of 18 mg/kg were prescribed for UFH group during the hospitalization. Increasing the dose continued until the PTT reached the therapeutic dose. In the LMWH group, first one mg/kg of LMWH was administered subcutaneously every 12 hours, and the dose was manipulated to target therapeutic range by monitoring of anti-factor Xa.

It is worth mentioning that the UFH of 20 000-30 000 units/day or 5000 IU every 4-6 hours in separated blouse doses was administrated subcutaneously to the control group according to the patient-physician shared decision either during the hospitalization period or after discharge from the hospital. However, LMWH group received out-patient subcutaneous administration. Moreover, 80 mg/kg of daily aspirin was administered to women with mitral metallic heart valve.

At the beginning of the pregnancy, patients had a baseline echocardiographic examination. Then, all patients were followed-up with regular echocardiography during every trimester. Appropriate actions were taken in the event of cardiovascular complications such as heart valve thrombosis, deteriorated cardiac symptoms, or other maternal complications. The patients referred to the maternal cardiac clinic were followed-up every two weeks up to 28 weeks and then weekly up to 36 weeks. Then, patients were admitted to hospital at 36 weeks pregnant. The birth weight, mode of delivery, gestational age at birth, and maternal complications such as bleeding, maternal death, thromboembolic complications, heart valve thrombosis, and thrombocytopenia as well as fetal complications such as fetal death, abortion, and retroplacental hematoma were recorded. It should be noted that the patients were followed up, and the data were recorded by a specialist unaware of the two groups studied.

### Statistical analysis

Finally, the SPSS software (version 22) was used to analyze the obtained data. The results were expressed as mean ± SD or n (%). To perform inferential statistics, Fisher’s exact test and independent samples *t* -tests were used. *P* value <0.05 was considered statistically significant.

## Results

In the present study, the UFH and LMWH groups consisted of 15 and 16 pregnant patients with the mean age of 32.67±9.11 and 5.81±31.50 years, respectively (*P* value=0.672). In both groups, the pregnant women had either mitral, aortic, or both mitral and aortic prosthetic valves. No significant difference was observed between the groups in terms of cardiovascular complications, weight, maternal age, and total number of pregnancies (*P* value> 0.05) (Table 1). In addition, as Table 2 indicates, there was no significant difference in the percentage of pregnancy complications between the two groups (*P* value > 0.05).

**Table 1:** Maternal characteristics

**Variables**	**UFH group (n=15)**	**LMWH group (n=16)**	***P *** **value**
Maternal age (year)	32.67±9.11	31.50±5.81	0.672
Weight (kg)	84.467±25.97	79.688±18.36	0.557
No. of total pregnancies	2.00±0.66	1.63±0.88	0.193
Position of metallic prosthetic valve			0.604
Mitral	13(86.7%)	12(75%)	
Aorta	1(6.7%)	3(18.8%)	
Mitral and aorta	1(6.7%)	1(6.3%)	

**Table 2:** Pregnancy outcomes

Outcomes	**UFH group (n = 15)**	**LMWH group (n = 16)**	***P*** **value**
Abortion	3 (20%)	1 (6.3%)	0.333
Live births			
Pre-term delivery	2 (16.7%)	0 (0%)	0.188
Full-term delivery	10 (83.3%)	15 (100%)	
EGA at birth (week)	37.40±1.95	37.19±1.22	0.735
Birth weight (g)	3470.12±351.79	3246.53±203.93	0.077
Mode of live birth delivery			
Vaginal delivery	8 (66.7%)	11 (73.3%)	0.706
Cesarean Section	4 (33.3%)	4 (26.7%)	
Early postpartum bleeding	0 (0%)	0 (0%)	-
Antepartum bleeding	0 (0%)	0 (0%)	-
Postpartum hematoma	3 (20%)	1 (6.3%)	0.333
Retroplacental hematoma	3 (20%)	1 (6.3%)	0.333
Thrombotic complications	3 (20%)	0 (0%)	0.101
Thrombocytopenia	0 (0%)	0 (0%)	-
Maternal death	0 (0%)	0 (0%)	-

Furthermore, it should be noted that about 30% of the patients receiving LMWH required a dose of more than 1 mg/kg every 12 hours to maintain the anti-factor Xa at the required therapeutic level; for example, one of the patients required a dose of 1.5 mg/kg every 12 hours.

Position of prosthetic valve (aortic or mitral) did not lead to any difference in the outcome of pregnancy, and there was not a significant difference in the position of prosthetic valve between two treatment groups.

## Discussion

The present study reported that three cases in the UFH group referred to the clinic with chest pain, dyspnea, and palpitations. The echocardiography study in these patients indicated prosthetic valve thrombosis, which was treated with thrombolytics (streptokinase). As the patients then experienced retroplacental hematoma with minor vaginal bleeding, they were monitored until the mentioned symptoms were resolved and the pregnancy continued in these patients. Two of the mentioned cases had full-term delivery while one case had pre-term delivery.

Moreover, none of complications such as early postpartum bleeding, antepartum bleeding, thrombocytopenia, and maternal death occurred in the present study.

It can be stated that although complications such as thrombocytopenia did not occur in the present study, heparin-induced thrombocytopenia (HIT) can be regarded as an adverse effect of heparin therapy that is a prothrombotic condition associated with characteristic platelet-activating antibodies. The mentioned complication is a reciprocal and variable immune response that results in the production of thrombin *in vivo* and is followed by the development of a hypercoagulable state and probably the onset of venous and arterial thrombosis. One of the effective factors on the occurrence of HIT is the type of heparin as well as the process of heparin (UFH and LMWH) production.^9,10^ In this regard, a previous study has reported that the incidence rate of this complication is significantly reduced in patients receiving LMWH.^11^

In contrast with the findings of the present study, Khader et al had reported adverse effects such as antepartum and postpartum bleeding in both UFH and LMWH groups.^12^ Moreover, the UFH group, in comparison with LMWH group, indicated the highest frequency of complications in the mentioned study. In our study, the rate of 20% was reported for each of the postpartum hematoma, retroplacental hematoma, and thrombotic complications in the UFH group, whereas only one case of postpartum hematoma and one case of retroplacental hematoma was recorded for the LMWH group. It should be mentioned that all the postpartum hematoma cases were mild, observed at the site of the rectus muscle, and were resolved in all cases conservatively with close observation.

In this regard, Regitz-Zagrosek et al proposed that LMWH was safe for the fetus; however, there were serious concerns about the safety of mothers as administration of LMWH was accompanied by 9% risk of heart valve thrombosis in the mentioned study.^13^

Later studies like Huxtable et al^14^ reported 7 out of 34 cases of thrombotic events and attributed all the thrombotic events to the sub-therapeutic anti-Xa levels or its non-compliance.^14^ In contrast, recent studies have indicated that LMWH is safe for pregnant women using anticoagulants when it is used with anti-Xa assey.^6,12^ Therefore, monitoring of the maximum anticoagulant activity using anti-Xa levels is currently recommended to ensure the safety of anticoagulation therapy with LMWH.^2^

Finally, abortion occurred in 20% and 6.3% of pregnancies in the UFH and LMWH groups, respectively. In fact, the rates of live birth in the UFH and LMWH groups were 80% and 93.7%, respectively. Similarly, the results of the study conducted by Khader et al^12^ revealed that 85% and 75% of pregnancies resulted in live births in the heparin and LMVH groups, respectively. In the mentioned study, only one intrauterine fetal death occurred in the LMVH group. Hence, in line with the findings of the present study, no significant difference was observed in the rate of live births between the two intervention groups.^8^ In a remarkable number of previous studies, the proportion of healthy babies delivered in both intervention groups was more than 55%.^15,16^

The major limitation of the present study was its small sample size that was unavoidable due to the low prevalence of this disease among pregnant women. The mentioned limitation restricts the generalizability of the findings. Hence, further studies are required to shed more light on this issue. Furthermore, a combination of two types of heparin in future studies will be more informative in this regard.

## Conclusion

According to the results of this study, the incidence rate of abortion in pregnant patients with metallic heart valve in the UFH group was higher than that of the LMWH group. Moreover, in general, the incidence rate of maternal complications in the UFH group was higher than that of the LMWH group, which can be attributed to the mother’s repeated failure to remember the use of the medication (at home). Moreover, the most frequent maternal and fetal complications were postpartum hematoma, retroplacental hematoma, and thrombotic complications that were less common in the LMWH group. In addition, as the frequency of laboratory evaluations of the anticoagulation during the treatment and also the frequency of daily administration of LMWH were lower in the LMWH group, the patients’ cooperation and satisfaction during the pregnancy werehigher than those of the UFH group. Given the mentioned advantage, it seems that further studies should be devoted to evaluate the pregnancy outcome in patients treated with LMWH vs. UFH to provide a more conclusive result.

## Competing interests

None declared.

## Ethical approval

Ethics Committee of Isfahan University of Medical Sciences. (IR.MUI.MED.REC.1397.362)

## Funding

None.
